# A Smarter Pavlovian Dog with Optically Modulated Associative Learning in an Organic Ferroelectric Neuromem

**DOI:** 10.34133/2021/9820502

**Published:** 2021-12-20

**Authors:** Mengjiao Pei, Changjin Wan, Qiong Chang, Jianhang Guo, Sai Jiang, Bowen Zhang, Xinran Wang, Yi Shi, Yun Li

**Affiliations:** ^1^National Laboratory of Solid-State Microstructures, School of Electronic Science and Engineering, Collaborative Innovation Center of Advanced Microstructures, Nanjing University, Nanjing 210093, China; ^2^School of Computing, Tokyo Institute of Technology, Tokyo 152-8550, Japan; ^3^School of Microelectronics and Control Engineering, Changzhou University, Changzhou 213164, China

## Abstract

Associative learning is a critical learning principle uniting discrete ideas and percepts to improve individuals' adaptability. However, enabling high tunability of the association processes as in biological counterparts and thus integration of multiple signals from the environment, ideally in a single device, is challenging. Here, we fabricate an organic ferroelectric neuromem capable of monadically implementing optically modulated associative learning. This approach couples the photogating effect at the interface with ferroelectric polarization switching, enabling highly tunable optical modulation of charge carriers. Our device acts as a smarter Pavlovian dog exhibiting adjustable associative learning with the training cycles tuned from thirteen to two. In particular, we obtain a large output difference (>10^3^), which is very similar to the all-or-nothing biological sensory/motor neuron spiking with decrementless conduction. As proof-of-concept demonstrations, photoferroelectric coupling-based applications in cryptography and logic gates are achieved in a single device, indicating compatibility with biological and digital data processing.

## 1. Introduction

The human brain outperforms digital computers in many complicated tasks, inspiring the replication of its functionality with artificial neurons and synapses towards excellent data processing capabilities similar to biological intelligence [1–5]. Brain-inspired computing possesses highly tunable native dynamics instead of the binary states in Boolean logic, which is beneficial for dealing with more complex real-world problems [6–10]. The modulation of synaptic plasticity in these devices follows the learning principles of the human brain. Associative learning is a learning principle in which ideas and experiences reinforce one another, which is critical to individuals during the extraction of the world logical structure for quick adaptation to the environment [11–14]. Classical associative learning is described by the Pavlovian dog, which starts to salivate to the ringing of a bell after a training process of feeding (unconditioned stimulus) and ringing a bell (neutral stimulus). After conditioning, a response can be triggered for both the unconditioned and neutral stimuli, with the latter becoming a conditioned stimulus. Recently, memristive devices or circuits have been designed to implement associative learning behaviour at the hardware level [15–24]. However, enabling high tunability of associative learning in electronic devices as in biological counterparts, which is key to further advancing associative learning hardware, is still challenging. In particular, integrating multiple signals to modulate the learning processes, which determines the adaptation of individuals to changing environments, should be increasingly taken into consideration. The tunability in the existing implementations is rather limited by merely changing the unconditioned and conditioned stimuli. In a bioinspired way, external influences from the surrounding environment, such as illumination, can also actively modulate the association processes. In addition, a large difference between neutral and conditioned responses is required to mimic the all-or-nothing biological neuron spiking, thus avoiding further construction of comparison circuits for precise differentiation. Ideally, this adjustable associative learning element with a large output difference should be based on a single device to provide the simplest geometry and reduce the power budget, delay, and number of circuit interconnections.

Here, an optically modulated organic neuromem with a two-terminal planar architecture using ferroelectric polymers and small-molecule semiconductors is fabricated. Based on coupling of the photogating effect at the interface and ferroelectric polarization switching, highly tunable optical modulation of charge carriers is achieved in a single device. A smarter Pavlovian dog is implemented, exhibiting adjustable associative learning with the training cycles tuned from thirteen to two under UV illumination. In addition, the energy consumption throughout the entire training process is only 84.9 to 9.4 *μ*J. In particular, a large difference between neutral and conditioned responses (>10^3^) is obtained through such learning processes in our monadic implementation, exhibiting great similarity to the all-or-nothing spiking and decrementless conduction in biological sensory/motor neurons. Encouragingly, based on such biologically comparable multiple signal processing based on photoferroelectric coupling, we extend our monadic associative learning device to applications in message enciphering and logic operations, closing the gap between highly tunable biological and digital data processing. Therefore, our results advance the development of associative learning hardware, laying a device foundation for brain-like systems towards artificial intelligence.

## 2. Results

### 2.1. Device Structure and Film Characterizations

**Figure 1:**
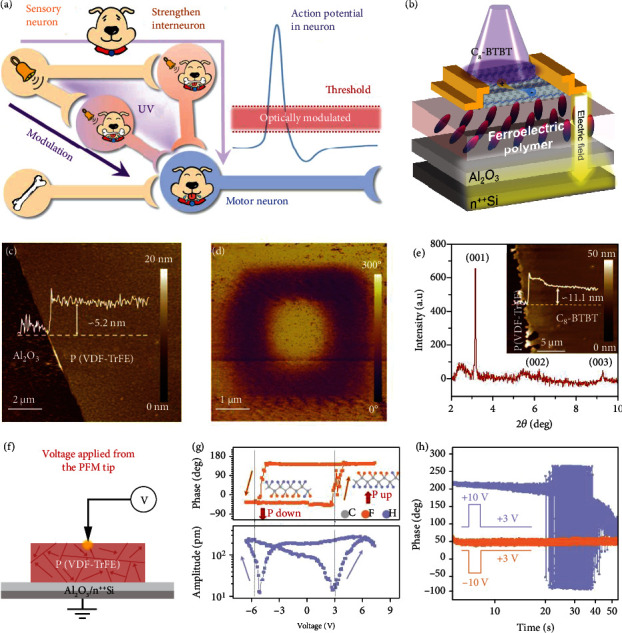
Device structure and characterizations of the functional films. (a) A typical neural mediating circuit layout for associative learning with the optical modulation. (b) Schematic illustration of the organic ferroelectric neuromem with a two-terminal planar architecture. (c) AFM height image and (d) PFM out-of-plane phase images of the ultrathin P(VDF-TrFE) film. (e) 2*θ* scan image taken from the XRD measurements of C_8_-BTBT on P(VDF-TrFE) films. The inset is the AFM height image of the C_8_-BTBT crystalline films. (f) Schematic illustration of the local PFM. (g) Local PFM phase and amplitude curves. The insets show the molecular structures corresponding to the two polarization states. (h) Phase versus testing time for an upward (purple) and a downward (orange) polarization orientation.

The transformation of the all-or-nothing responses in motor neurons to the same stimulus before and after training is the key mechanism involved in associative learning of the biological nervous system. A typical neural mediating circuit layout for associative learning is shown in Figure 1(a), which includes two sensory neurons (S), an interneuron (I), and a motor neuron (M). The neutral stimulus (NS) applied to a sensory neuron cannot induce an action potential in the motor neuron until an enhanced interconnection with the unconditioned stimulus (US) is established via the interneuron. The state of the interneuron critically determines the establishment of the association, whose electronic version can correspond to ferroelectric polarization switching in devices. Therefore, we fabricated an organic ferroelectric neuromem with a two-terminal planar architecture using the ultrathin polymer Poly(vinylidene fluoride-cotrifluoroethylene) (P(VDF-TrFE)) and small-molecule semiconductor dioctylbenzothienobenzothiophene (C_8_-BTBT) as the functional layers (Figure 1(b) and fig. S1). During device fabrication, two 100 nm gold electrodes with dimensions of 30 × 100 *μ*m^2^ were directly transferred onto the surface of functional films. A major advantage of ferroelectric polymers is their compatibility with any substrate at low temperatures [25, 26]. A highly doped Si substrate coated with ~5 nm Al_2_O_3_ was prepared for the deposition of P(VDF-TrFE) (70 : 30 mole ratio) from a mixture of *N*,*N*-dimethylformamide (DMF) and the antisolvent *p*-anisaldehyde (~5 mg mL^−1^) at a 0.5 wt. % concentration. With the antisolvent-assisted approach and treatment at 40°C on a hot plate for 10 min to increase the crystallinity, the deposited P(VDF-TrFE) film was clearly distinguished on the substrate (fig. S2), and its morphological properties were further studied by atomic force microscopy (AFM) measurements. The P(VDF-TrFE) layer was as thin as ~3.3 ± 0.2 nm, exhibiting a smooth surface with a root-mean-square (RMS) roughness of ~0.73 nm (Figure 1(c)). The AFM measurements were performed at different areas selected randomly, exhibiting a RMS roughness of less than 1 nm (fig. S3).

On the ultrasmooth ferroelectric film, a 5 × 5 *μ*m^2^ square with upward polarization was defined by scanning the grounded AFM tip with voltages of ±9 V applied to the bottom electrode (fig. S4). A clear 180° phase shift was observed relative to the pristine P(VDF-TrFE) background with a homogeneous downward polarization (Figure 1(d)). The ferroelectricity originates from the crystalline phase of P(VDF-TrFE), whose grains can be clearly observed in the AFM morphology characterizations. In addition, the ultrathin crystalline P(VDF-TrFE) with smooth surface allows a deposition of a highly crystalline small-molecule semiconductor C_8_-BTBT with the thickness of ~11.1 nm (Figure 1(e)). Furthermore, the ferroelectric properties were explored via a local probe in a piezoresponse force microscopy (PFM) setup (Figure 1(f)). Voltages of variable amplitude within ±7 V were applied to induce local domain switching. We observed a clear anticlockwise hysteresis with a 180° phase contrast and a typical butterfly-like amplitude shape, revealing that the molecular dipole moments at different polarization states had nearly antiparallel orientations (Figure 1(g)). Thus, structural deformation and polarization switching of the ultrathin P(VDF-TrFE) film are simultaneously accomplished by the PFM tip-generated poling field. The coercive voltages at which ferroelectric polarization reversals occur are approximately +2.4 and −5.4 V. Generally, as the thickness of a ferroelectric film shrinks, the depolarization field, which arises from the surface-bound charges, becomes stronger [27, 28]. Hence, time-dependent measurements imaging the evolution of the piezoelectric property, related to the reorientation of the polarization states, were performed. The ultrathin P(VDF-TrFE) exhibited a stable spontaneous downward polarization state, whereas the upward polarization showed polarization relaxation (Figure 1(h)).

### 2.2. Ferroelectric Polarization Determined Optoelectronic Devices

**Figure 2:**
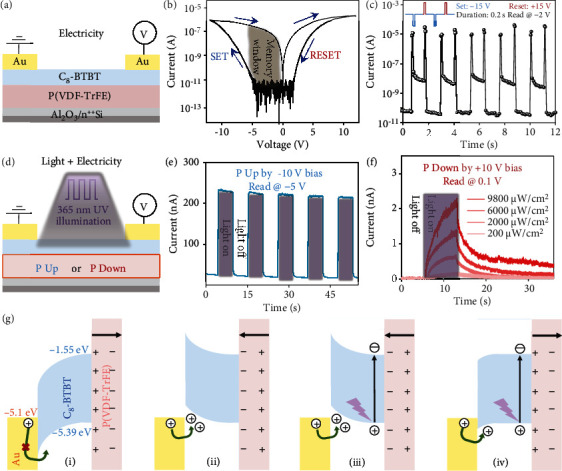
Polarization switching and photoelectric coupling in devices. (a) Schematic illustration of the electrical measurements. (b) Typical I-V curves of the device. The set and reset processes are shown as arrows. The channel length and channel width of the device are 75 and 5 *μ*m, respectively. (c) Repeated erase/read/program/read sequence with voltages of 15 V/–1 V/–15 V/–1 V, respectively. (d) Schematic illustration of photoelectrical measurements in the upward and downward polarization states of P(VDF-TrFE) films. (e) Photoswitching behaviour of devices at *V* = −5 V after polarizing them up by a −10 V bias (365 nm UV light source; light intensity: 200 *μ*W cm^−2^; and frequency: 0.1 Hz). (f) Synaptic behaviour of devices at *V* = 0.1 V after polarizing them down by a +10 V bias. The current is triggered by various light intensities on the devices. (g) Schematic illustration of the band diagram.

Considering the ultrathin functional films, application of a voltage between the two planar electrodes can also efficiently realize field modulation [29, 30]. Hence, during all the subsequent electrical measurements, one Au electrode was grounded, and an external bias was applied to the other electrode (Figure 2(a)). The upward and downward ferroelectric polarization directions can be well switched by applying different voltages, assisting efficient accumulation and depletion of carriers in the organic ferroelectric neuromem with a two-terminal planar architecture. The processes are similar to those in ferroelectric field-effect transistor (FET) memories operated by a gate voltage with three terminals [31–35]. The main difference is that the external electric field is transmitted through the ultrathin functional layers, subsequently inducing ferroelectric polarization switching. For a voltage sweep from +12 V to –12 V (sweeping directions indicated by arrows), the resistivity of C_8_-BTBT changed from the high-resistance (OFF) state to the low-resistance (ON) state and back to the OFF state, yielding a current hysteresis (Figure 2(b)). This switching behaviour was reproducible during the subsequent series of voltage sweeps. A series of memory cycles with repeated voltage pulses of +15 V, –1 V, –15 V, and –1 V were used as the resetting, reading, setting, and reading operations, respectively (Figure 2(c)). A nondestructive read state with an on/off ratio of over 1000 was achieved, which can also be inferred from the current hysteresis loop, as shown in the typical *I-V* curves of the device. In addition, after setting and resetting operations by applying –12 V and +12 V, respectively, the *I–V* curves in the low-voltage range of ±2 V clearly revealed two different polarization states. Besides, to evaluate the stability of our devices, retention measurements were performed, showing that the currents in on and off states maintained an on/off ratio of >10 during a prolonged time scale of 2500 s (fig. S5).

Based on the well-switched ferroelectric polarization, we further studied the optoelectronic performance of our devices (Figure 2(d)). Considering that the C_8_-BTBT layer works at the ultraviolet range with a maximum absorption peak value of ~358 nm, a 365 nm UV light source was applied (fig. S6). When the ferroelectric polarization was upward, the current in the active layers rapidly increased under UV illumination with photon energy above *E*_g_ due to the photogenerated carriers (Figure 2(e)). After the UV light was terminated, the current returned to the initial dark value due to the absence of additional photogenerated carriers. The rise (*t*_*r*_) and decay (*t*_*d*_) times, defined as the interval for the response to rise/decay from 10%/90% to 90%/10% of the drain current under light illumination, were estimated to be ~134.7 and 148.7 ms, respectively. Interestingly, when the ferroelectric dipoles were switched downward, the same device acted as a light-stimulated artificial synapse (Figure 2(f)). Similarly, an increment in the channel conductivity appeared when illuminated. Then, the current underwent a process of gradual decay after the light illumination was removed, which was clearly distinct from the quickly reduced photocurrent under upward polarization. In addition to the light intensity, the performance of a light-stimulated organic artificial synapse was further characterized by changing the number, frequency, and time interval of pulsed light stimuli (fig. S7). The ability to change the strength of the synaptic connections via a single repetitive stimulus corresponds to the biological basis of nonassociative learning behaviour in human brains.

In addition, the relationships between the direction of the ferroelectric polarization and different photoelectric properties were further confirmed in a three-terminal transistor with the bottom-gate top-contact structure (fig. S8). The operation mode of polarization switching and photoferroelectric coupling in our devices is schematically illustrated in Figure 2(g). When the polarization direction of ferroelectric dipoles is (i) upward or (ii) downward, holes accumulate or deplete in the active layers, respectively. Under light illumination, the C_8_-BTBT films absorb incident photons and then generate a vast number of excitons, which can be separated by the strong polarization-induced localized field at the P(VDF-TrFE)/C_8_-BTBT interface. Then, the external electric field drives unpaired holes to quickly migrate in the conducting channel, resulting in an increment in the conductance in both polarization states (iii and iv). The unpaired electrons are quickly collected by the cathode under upward polarization, while they tend to be captured by the interfacial traps under the influence of downward dipoles and can then be slowly released.

### 2.3. A smarter Pavlovian Dog with Optically Modulated Associative Learning

Pavlovian associative learning behaviours have been mimicked in many electronic versions, such as memristors, electrochemical transistors, and circuits, to improve the learning efficiency and increase the integration intensity of brain-inspired computing systems. Notably, multi-input modulation during training processes, revealing the rich dynamics and complex computational tasks in biological systems, should be considered more for further development of associative learning devices.

**Figure 3:**
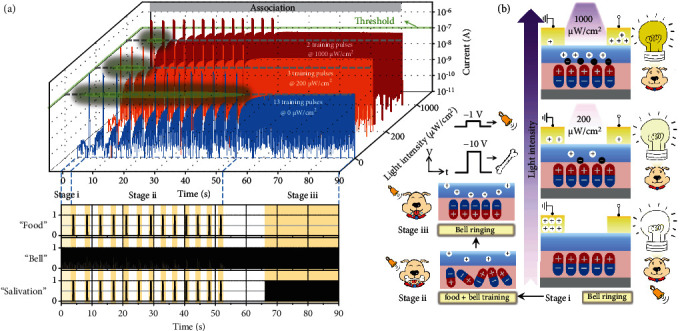
Optically adjustable associative learning processes. (a) Dynamic associative learning process in the same device under different illumination conditions (0, 200, and 1000 *μ*W/cm^2^). A large voltage spike of −10 V is deemed “food,” and a small voltage spike of −1 V is deemed “bell.” The current is measured and compared to the neuron's threshold of 10^−7^ A. (b) Schematic of the three stages in the learning processes and the corresponding electrostatic properties at the semiconductor/ferroelectric interfaces. Stage i of “ring bell” under different illumination conditions indicates a smarter Pavlovian dog with increasing light intensity.

First, we mimicked an electronic Pavlovian dog by taking advantage of the polarization dynamics in ultrathin P(VDF-TrFE). During the training process, food is a US of a −10 V spike that produces an unconditioned response (UR), i.e., salivation, while an NS of −1 V, i.e., bell ringing, causes a neutral response (NR). Before training, the UR of ~10 *μ*A was approximately five orders of magnitude higher than the NR, largely increasing the distinction between the two stimuli (fig. S9). Upward polarization in the ultrathin P(VDF-TrFE) was induced by the US of −10 V above the coercive voltage, inducing charge accumulation in the active layers. In stage i of the training process, the NS of −1 V was not enough to change the stable spontaneous downward polarization in P(VDF-TrFE), leading to a low response current of ~10^−10^ A (Figure 3(a)). When the paired “bell” (NS) and “food” (US) signals were repeatedly applied with an interval of 250 ms in stage ii, the polarization state gradually turned upward, with an increase in the output current for the same NS after every US. The value of 10^−7^ A was defined as the threshold for a “salivation” response. After applying 13 US/NS pairs, the ferroelectric polarization was completely switched to the upward direction, assisting a large output current of over ~10^−7^ A when applying −1 V alone after 30 s (stage iii). An association was established between the NS and US, in which the NS produces a similar “salivation” response as the US and can be called a conditioned stimulus (CS). The highly distinguishable difference between the NR and CR is larger than 10^3^, which is a record high value in the literature, yielding a significant similarity to the all-or-nothing biological neuron spiking (table S1).

In particular, the organic semiconducting films in the proposed ferroelectric neuromem can act as both light-sensitive and neuromorphic readout elements [36–38]. Hence, under UV light illumination, a smarter Pavlovian dog can be realized with an optically modulated learning procedure benefiting from efficient photoferroelectric coupling. During the learning process, i.e., gradual polarization reversal and formation of conducting channels, electron trapping at the P(VDF-TrFE)/C_8_-BTBT interface also contributes to modulating the conductivity of the semiconductors. In the same device under different illumination conditions (0, 200, and 1000 *μ*W/cm^2^), the output currents for the CS (the initial NS of a small voltage spike of −1 V) increased faster to the dotted line (completion of training) with increasing light intensity. For further quantitative analysis, the output currents for the CS depending on the number of US (−10 V) during the training process were extracted (fig. S10). The training results under different light intensities showed the largest difference in the first few pulses. During the training of the first four US/NS pairs, the slope of the output current for the CS curve depending on the number of US significantly increased from ~9.2 × 10^−10^ A to ~6.9 × 10^−8^ A. The rapid increase in the output current with strengthened light intensity is highly related to the properties of the P(VDF-TrFE)/C_8_-BTBT interface. Particularly, at the beginning of the training processes, the charge carriers were depleted in the conducting channels due to the spontaneous downward polarization of the ultrathin P(VDF-TrFE) on Al_2_O_3_. The stable and preferential state was beneficial to plasticity enhancement in light-stimulated artificial synapses (Figure 3(b)). Hence, more excitons were continuously generated and separated under UV illumination with increased intensity. Then, more unpaired electrons from these photogenerated excitons were trapped at the P(VDF-TrFE)/C_8_-BTBT interface when continued illumination was applied. The photogating effect generated by trapped electrons could be a supplement to the upward ferroelectric polarization. It well assisted the accelerated accumulation in organic conducting channels, corresponding to a smarter Pavlovian dog with the unique ability to learn more quickly during the training procedures. In addition, we estimated the energy consumption throughout the entire training process of our organic ferroelectric neuromem, which can be expressed as

(1) $$\begin{array}{c} E=\int_{0}^{t_{\text{th}}}V\times I\;dt, \end{array}$$

where *t*_th_ is the time of the current reaches the threshold for a “salivation” response (10^−7^ A), *V* is the voltage applied to the device during the training process, and *I* is the current in the device during training. The energy consumption throughout the entire training process is only 9.4 (2 training pulses) to 84.9 *μ*J (13 training pulses). Notably, benefiting from the efficient photoferroelectric coupling in our device, a monadic implementation is realized without complicated electrical interconnects or changing of the preset input sequences. Therefore, an organic ferroelectric neuromem with a two-terminal planar architecture is a fascinating platform for adjustable associative learning, strengthening the universality of field modulation of charge carriers through photoferroelectric coupling for biomimetic signal-processing functional elements [39–41].

### 2.4. Cryptographical and Logical Applications

**Figure 4:**
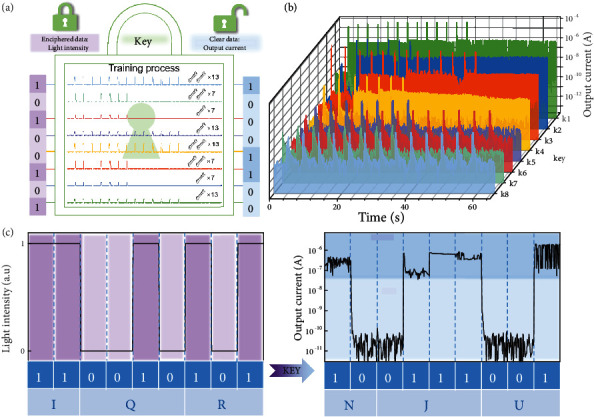
Demonstration of a cryptographical application based on Pavlovian associative learning. (a) Setting of enciphered data, clear data, and key in our devices. (b) Eight selected training processes in the device. (c) Decryption of the Morse code characters of “NJU.”

The efficient photoferroelectric coupling during the associative learning process shows the tunable establishment of a relationship between two objects, which is intrinsically similar to the property of the key in cryptography. Specifically, the transformation of enciphered data to clear data with the corresponding key is also a process in which event A connects to event B. Hence, the proposed optically modulated organic ferroelectric neuromem is potentially attractive for hardware security applications. A common encryption method for electronic devices is to display effective information under a specific wavelength of light based on the optical characteristics of the material [42–46]. Once the material is prepared with the selected decrypted light at the corresponding wavelength, the instant tunability of the transmitted information is limited, which can be regarded as a static encryption method. Nevertheless, considering that Pavlovian associative learning behaviour is intrinsically a dynamic procedure, we can change the information we want to transmit at any time in the same device through external photoelectric signals. Moreover, the unique optical modulation based on photoferroelectric coupling in devices increases the complexity of the key, thus greatly increasing the difficulty of decoding. We designed a coding method based on the device characteristics. As shown in Figure 4(a), the enciphered data are a 0-1 signal distinguished by the intensity of 365 nm UV illumination (0: 2000 *μ*W/cm^2^ and 1: 6000 *μ*W/cm^2^). The clear data are defined as a binary signal indicating whether the output current varies by three orders of magnitude. The key is set to an array-like (*x* and *y*), in which “*x*” represents whether dogs are subjected to unconditioned stimuli (1: yes and 0: no) and “*y*” represents whether the number of unconditioned stimuli can complete the training of the dog (1: yes and 0: no). The enciphered data, clear data, and corresponding keys between them are listed. When we adjust the number of pulses and the intensity of light illumination, eight different combinations can be obtained. Moreover, a mapping relationship between the code and the keys without overlapping or misplacement of information transmission is shown in Figure 4(b). Therefore, we defined the long and short signals in Morse code as the high and low currents, respectively. Then, decryption of the simple Morse code characters of IQR (--▪▪-▪-▪-) and NJU (-▪▪---▪▪-) was demonstrated (Figure 4(c)). These results provide an interesting concept for developing out-of-the-box security based on associative learning behaviours by introducing novel device design and materials.

**Figure 5:**
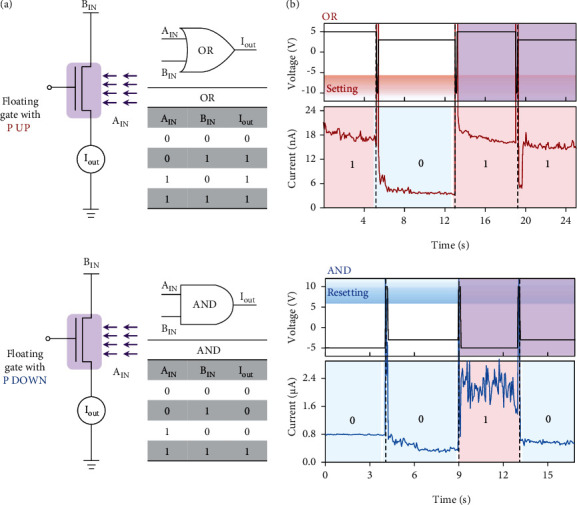
Logic photoelectric devices based on photoferroelectric coupling. (a) Schematic diagram of the logic photoelectric switch (“1”: a high input voltage with an amplitude of 5 V, a large illumination intensity (9600 *μ*W/cm^2^), and a high output current and “0”: a low input voltage with an amplitude of 3 V, a small illumination intensity, and a low output current). (b) Current switching behaviour obtained by manipulating the input voltage under continuous illumination with different intensities. The current spikes are due to applying the set (–10 V) or reset (+10 V) voltages to ensure that the polarization direction is up or down, respectively.

In addition to regulation of dynamic learning processes, efficient photoferroelectric coupling in a single device also benefits optoelectronic Boolean logic applications. Generally, the logic OR and AND functions are the two basic logic gates, whose corresponding NOR and NAND logic functions can be used to construct a complete logic system [47]. In traditional circuit design, more than six transistors are needed to complete the basic logic gate operations. Nevertheless, in a single device, optical and electrical signals can be utilized as the two inputs with simultaneous modulation of the conducting channel by illumination and ferroelectric polarization. As shown in Figure 5(a), *A*_IN_ is defined by the optical signals, where UV illumination (9600 *μ*W/cm^2^) represents 1 and no light represents 0. *B*_IN_ is the amplitude of the voltage pulses, where the high voltage amplitudes represent 1 and the low voltage amplitudes represent 0. In addition, to ensure an upward or downward ferroelectric polarization before each logical operation, set (−10 V) or reset (+10 V) voltages were applied in advance, respectively. With efficient modulation of the photoelectric properties of the semiconducting channels by altering the critical value of the required pulse and light operations, OR/AND gate operations were realized in the same device (Figure 5(b)). The results clearly showed that the output of the OR gate operation was 0 (low) only when both the inputs *A*_IN_ and *B*_IN_ were 0; otherwise, it was 1 (high) with a threefold current difference. The output of the AND gate operation was 1 (high) only when both the inputs *A*_IN_ and *B*_IN_ were 1; otherwise, it was 0 (low), indicating the reconfigurability of the devices.

## 3. Discussion

In conclusion, we have mimicked optically modulated monadic associative learning behaviours using an organic ferroelectric neuromem with a two-terminal planar architecture. The approach couples the photogating effect at the interface and ferroelectric polarization switching, allowing highly tunable optical modulation of charge carriers in a single device. A smarter Pavlovian dog exhibiting associative learning is implemented with the training cycles tuned from thirteen to two and low energy consumption throughout the entire training processes. In particular, we achieved a large output difference of >10^3^ during such adjustable learning processes in our monadic implementation, closely corresponding to the all-or-nothing biological neuron spiking with decrementless conduction. As proof-of-concept demonstrations, applications in message enciphering, and logic operations are achieved in a single device, indicating compatibility with biological and digital data processing. Our results demonstrate the possibility of progressing associative learning hardware towards brain-inspired artificial intelligence.

## 4. Materials and Methods

### 4.1. Deposition of P(VDF-TrFE) and C_8_-BTBT Crystals

A highly doped Si substrate coated with ~5 nm Al_2_O_3_ by atomic layer deposition was sequentially cleaned in an ultrasonic bath with acetone, isopropanol, and deionized water for 10 min each. Then, P(VDF-TrFE) (70 : 30 mole ratio, purchased from Solvay, Inc., France) was dissolved in a mixture of DMF and the antisolvent *p*-anisaldehyde (~5 mg mL^−1^) at a 0.5 wt. % concentration. A droplet of the P(VDF-TrFE) solution was then drop-cast onto the substrate; a mechanical pump with a pumping speed of ~7 L min^−1^ was used to vent air through a pipe positioned ~1 mm from the upper surface of the droplet in a glove box under high-purity N_2_ conditions. As the solution edge moved, deposited P(VDF-TrFE) films could be obtained after the solvent evaporated at room temperature and clearly distinguished on the substrate and were then treated at 40°C on a hot plate for 10 min to increase the crystallinity. The small-molecule semiconductor C_8_-BTBT (Sigma-Aldrich) was dissolved in a solvent mixture with anisole (Sigma-Aldrich) (0.5 wt. %). C_8_-BTBT crystals were deposited on the surface of P(VDF-TrFE) as the active layers under ambient conditions by spin-coating at 500 rpm for 5 s and 2000 rpm for 60 s.

### 4.2. Device Fabrication

Patterned Au films with a thickness of 100 nm and Au pads with dimensions of 30 × 100 *μ*m^2^ were thermally evaporated under a deposition speed of 0.2 Å s^−1^. Two Au pads were subsequently transferred to the top of the C_8_-BTBT crystal to form the source and drain electrodes. The channel width and length of these Fe-OFETs were 75 and 5 *μ*m, respectively.

### 4.3. Electrical Characterizations

A KEYSIGHT B1500A semiconductor device analyzer was used for the electrical characterizations of our devices under ambient conditions.

### 4.4. AFM and PFM Measurements

Regular AFM characterizations were performed on a scanning probe microscope (SPA-400) controlled by an SPI 4000 probe station (Seiko Instruments, Inc.). The piezoelectric hysteresis loop and domain piezoelectric behaviour measurements were performed with an Asylum Research Cypher scanning probe microscope (Asylum Research, Oxford Instruments, China) using Nanosensors PPP-EFM chromium/platinum-iridium (Cr/Pt-Ir)-coated silicon cantilevers (radius of ~25 nm).

#### 4.4.1. XRD Measurements

Out-of-plane XRD was performed by a Rigaku SmartLab X-ray diffractometer operated at a 3 kW X-ray power to assess the crystalline properties of the P(VDF-TrFE) and C_8_-BTBT crystals.

#### 4.4.2. UV Absorption Measurements

We obtained the absorbance spectra of the C_8_-BTBT films on quartz by using Shimadzu UV3600 (UV-vis-NIR) spectrometer in the range of 300–700 nm.

## Data Availability

All data that support the findings of this study are available from the corresponding author upon reasonable request.

## Supplementary Materials

Supplementary Materials Fig. S1: molecular structures. Fig. S2: optical microscopy of the ultrathin P(VDF-TrFE). Fig. S3: AFM images of the ultrathin crystalline P(VDF-TrFE) film on Al2O3. Fig. S4: out-of-plane PFM measurements. Fig. S5: the memory performance of the two-terminal planar organic ferroelectric devices. Fig. S6: absorption spectra of C_8_-BTBT films on quartz. Fig. S7: an optically modulated organic artificial synapse under the downward polarization state of P(VDF-TrFE). Fig. S8: the optoelectronic performance of the three-terminal organic ferroelectric devices. Fig. S9: unconditioned stimulus (US) and neutral stimulus (NS) and their current responses. Fig. S10: quantitative analysis. Note 1: performance of a light-stimulated organic artificial synapse. Table S1: comparison with the electronic Pavlovian dogs in literature.

## References

[1] Roy K., Jaiswal A., Panda P. (2019). Towards spike-based machine intelligence with neuromorphic computing. *Nature*.

[2] Yang J., Wang R., Ren Y. (2020). Neuromorphic engineering: from biological to spike-based hardware nervous systems. *Advanced Materials*.

[3] Kumar S., Williams R. S., Wang Z. (2020). Third-order nanocircuit elements for neuromorphic engineering. *Nature*.

[4] Upadhyay N. K., Jiang H., Wang Z., Asapu S., Xia Q., Joshua Yang J. (2019). Emerging memory devices for neuromorphic computing. *Advanced Materials Technologies*.

[5] Wang J., Zhuge F. (2019). Memristive synapses for brain-inspired computing. *Advanced Materials Technologies*.

[6] Roe D. G., Kim S., Choi Y. Y. (2021). Biologically plausible artificial synaptic array: replicating Ebbinghaus’ memory curve with selective attention. *Advanced Materials*.

[7] Kim M., Lee J. (2020). Synergistic improvement of long-term plasticity in photonic synapses using ferroelectric polarization in Hafnia-based oxide-semiconductor transistors. *Advanced Materials*.

[8] Lee T. H., Hwang H. G., Woo J. U., Kim D. H., Kim T. W., Nahm S. (2018). Synaptic plasticity and metaplasticity of biological synapse realized in a KNbO3Memristor for application to artificial synapse. *ACS Applied Materials & Interfaces*.

[9] Erokhin V., Berzina T., Camorani P. (2011). Material memristive device circuits with synaptic plasticity: learning and memory. *BioNanoScience*.

[10] Yoon C., Lee J. H., Lee S. (2017). Synaptic plasticity selectively activated by polarization-dependent energy-efficient ion migration in an ultrathin ferroelectric tunnel junction. *Nano Letters*.

[11] Walle A., Hübner R., Druey M. D. (2021). Value associations modulate visual attention and response selection. *Frontiers in Psychology*.

[12] Theeuwes J. (2019). Goal-driven, stimulus-driven, and history-driven selection. *Current Opinion in Psychology*.

[13] Bucker B., Theeuwes J. (2018). Stimulus-driven and goal-driven effects on Pavlovian associative reward learning. *Visual Cognition*.

[14] Thompson R. F., Bao S., Chen L. (1997). Associative learning. *International Review of Neurobiology*.

[15] Wang L., Zou H. (2020). A new emotion model of associative memory neural network based on memristor. *Neurocomputing*.

[16] Li Y., Xu L., Zhong Y.-P. (2015). Associative learning with temporal contiguity in a memristive circuit for large-scale neuromorphic networks. *Advanced Electronic Materials*.

[17] Pei Y., Zhou Z., Chen A. P., Chen J., Yan X. (2020). A carbon-based memristor design for associative learning activities and neuromorphic computing. *Nanoscale*.

[18] Maier P., Hartmann F., Emmerling M. (2017). Associative learning with Y-shaped floating gate transistors operated in memristive modes. *Applied Physics Letters*.

[19] Wang L., Li H., Duan S., Huang T., Wang H. (2016). Pavlov associative memory in a memristive neural network and its circuit implementation. *Neurocomputing*.

[20] Bichler O., Zhao W., Alibart F. (2013). Pavlov’s dog associative learning demonstrated on synaptic-like organic transistors. *Neural Computation*.

[21] Yan M., Zhu Q., Wang S. (2021). Ferroelectric synaptic transistor network for associative memory. *Advanced Electronic Materials*.

[22] Zhou M., Wang L., Duan S., Lu H., Tang H., Wang Z. (2019). An Improved Memristor-Based Associative Memory Circuit for Full-Function Pavlov Experiment. *Advances in Neural Networks – ISNN 2019. ISNN 2019*.

[23] Sun J., Han G., Zeng Z., Wang Y. (2019). Memristor-based neural network circuit of full-function Pavlov associative memory with time delay and variable learning rate. *IEEE Transactions on Cybernetics*.

[24] Shang M., Wang X. (2020). A memristor-based circuit design for generalization and differentiation on Pavlov associative memory. *Neurocomputing*.

[25] Qian J., Jiang S., Wang Q. (2018). Unveiling the piezoelectric nature of polar *α*-phase P(VDF-TrFE) at quasi- two-dimensional limit. *Scientific Reports*.

[26] Qian J., Jiang S., Wang Q. (2018). Temperature dependence of piezo- and ferroelectricity in ultrathin P(VDF–TrFE) films. *RSC Advances*.

[27] Liu G., Chen J., Lichtensteiger C. (2016). Positive effect of an internal depolarization field in ultrathin epitaxial ferroelectric films. *Advanced Electronic Materials*.

[28] Guan Z., Hu H., Shen X. (2020). Recent progress in two-dimensional ferroelectric materials. *Advanced Electronic Materials*.

[29] Tran M. D., Kim H., Kim J. S. (2019). Two-terminal multibit optical memory via van der Waals heterostructure. *Advanced Materials*.

[30] Vu Q. A., Shin Y. S., Kim Y. R. (2016). Two-terminal floating-gate memory with van der Waals heterostructures for ultrahigh on/off ratio. *Nature Communications*.

[31] Song L., Wang Y., Gao Q. (2017). Speed up ferroelectric organic transistor memories by using two-dimensional molecular crystalline semiconductors. *ACS Applied Materials & Interfaces*.

[32] Pei M., Qian J., Jiang S. (2019). PJ-level energy-consuming, low-voltage ferroelectric organic field-effect transistor memories. *The Journal of Physical Chemistry Letters*.

[33] Xu M., Xiang L., Xu T., Wang W., Xie W., Zhou D. (2017). Low-voltage operating flexible ferroelectric organic field-effect transistor nonvolatile memory with a vertical phase separation P(VDF-TrFE-CTFE)/PS dielectric. *Applied Physics Letters*.

[34] Xiang L., Wang W., Xie W. (2016). Achieving high mobility, low-voltage operating organic field-effect transistor nonvolatile memory by an ultraviolet-ozone treating ferroelectric terpolymer. *Scientific Reports*.

[35] Wang Y., Kizu T., Song L. (2016). High-performance non-volatile field-effect transistor memories using an amorphous oxide semiconductor and ferroelectric polymer. *Journal of Materials Chemistry C*.

[36] Liu J., Jiang L., Shi J. (2020). Relieving the photosensitivity of organic field-effect transistors. *Advanced Materials*.

[37] Shi Y., Jiang L., Liu J. (2018). Bottom-up growth of n-type monolayer molecular crystals on polymeric substrate for optoelectronic device applications. *Nature Communications*.

[38] Fan Y., Liu J., Hu W., Liu Y., Jiang L. (2020). The effect of thickness on the optoelectronic properties of organic field-effect transistors: towards molecular crystals at monolayer limit. *Journal of Materials Chemistry C*.

[39] He Z., Shen H., Ye D. (2021). An organic transistor with light intensity-dependent active photoadaptation. *Nature Electronics*.

[40] Yang C., Qian J., Jiang S. (2020). An optically modulated organic Schottky-barrier planar-diode-based artificial synapse. *Advanced Optical Materials*.

[41] Zang Y., Shen H., Huang D., Di C.-A., Zhu D. (2017). A dual-organic-transistor-based tactile-perception system with signal-processing functionality. *Advanced Materials*.

[42] Chen H., Lv L., Wei Y. (2021). Self-powered flexible artificial synapse for near-infrared light detection. *Cell Reports Physical Science*.

[43] Zhou Y., Han S.-T., Chen X., Wang F., Tang Y. B., Roy V. A. L. (2014). An upconverted photonic nonvolatile memory. *Nature Communications*.

[44] Gu L., Shi H., Bian L. (2019). Colour-tunable ultra-long organic phosphorescence of a single-component molecular crystal. *Nature Photonics*.

[45] Wang Z., Meng F., Zhang S., Meng Y., Wu S., Tang B. (2020). Robust, portable, and specific water-response silk film with noniridescent pattern encryption for information security. *ACS Applied Materials & Interfaces*.

[46] Su Y., Phua S. Z. F., Li Y. (2018). Ultralong room temperature phosphorescence from amorphous organic materials toward confidential information encryption and decryption. *Science Advances*.

[47] Liu C., Chen H., Hou X. (2019). Small footprint transistor architecture for photoswitching logic and in situ memory. *Nature Nanotechnology*.

