# Supplementary material for: A Smarter Pavlovian Dog with Optically Modulated Associative Learning in an Organic Ferroelectric Neuromem
Source: Research (Wash D C). 2021 Dec 20;2021:9820502. doi: 10.34133/2021/9820502 (PMC8715308; doi:10.34133/2021/9820502)
Supplement: Supplementary Materials — Fig. S1: molecular structures. Fig. S2: optical microscopy of the ultrathin P(VDF-TrFE). Fig. S3: AFM images of the ultrathin crystalline P(VDF-TrFE) film on Al2O3. Fig. S4: out-of-plane PFM measurements. Fig. S5: the memory performance of the two-terminal planar organic ferroelectric devices. Fig. S6: absorption spectra of C8-BTBT films on quartz. Fig. S7: an optically modulated organic artificial synapse under the downward polarization state of P(VDF-TrFE). Fig. S8: the optoelectronic performance of the three-terminal organic ferroelectric devices. Fig. S9: unconditioned stimulus (US) and neutral stimulus (NS) and their current responses. Fig. S10: quantitative analysis. Note 1: performance of a light-stimulated organic artificial synapse. Table S1: comparison with the electronic Pavlovian dogs in literature. [file 9820502.f1.docx]

Supplementary Materials

Title

A Smarter Pavlovian Dog with Optically Modulated Associative Learning in an Organic Ferroelectric Neuromem

**Authors**

Mengjiao Pei^1^†, Changjin Wan^1^†, Qiong Chang^2^†, Jianhang Guo^1^, Sai Jiang^3^, Bowen Zhang^1^, Xinran Wang^1^, Yi Shi^1*^, and Yun Li^1*^

**Affiliations**

^1^National Laboratory of Solid-State Microstructures, School of Electronic Science and Engineering, Collaborative Innovation Center of Advanced Microstructures, Nanjing University, Nanjing 210093, P. R. China.

^2^School of Computing, Tokyo Institute of Technology, Tokyo 152-8550, Japan.

^3^School of Microelectronics and Control Engineering, Changzhou University, Changzhou 213164, P. R. China.

* Correspondence should be addressed to Yi Shi; yshi@nju.edu.cn and Yun Li; yli@nju.edu.cn

† These authors contributed equally to this work.


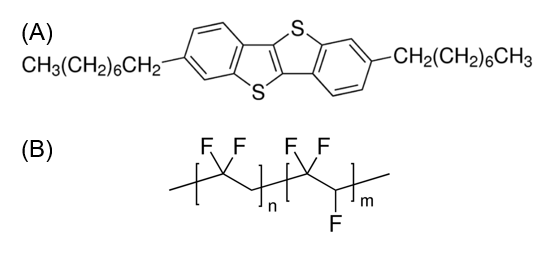


***Figure S1. Molecular structures.*** *(A) C_8_-BTBT. (B) P(VDF-TrFE).*


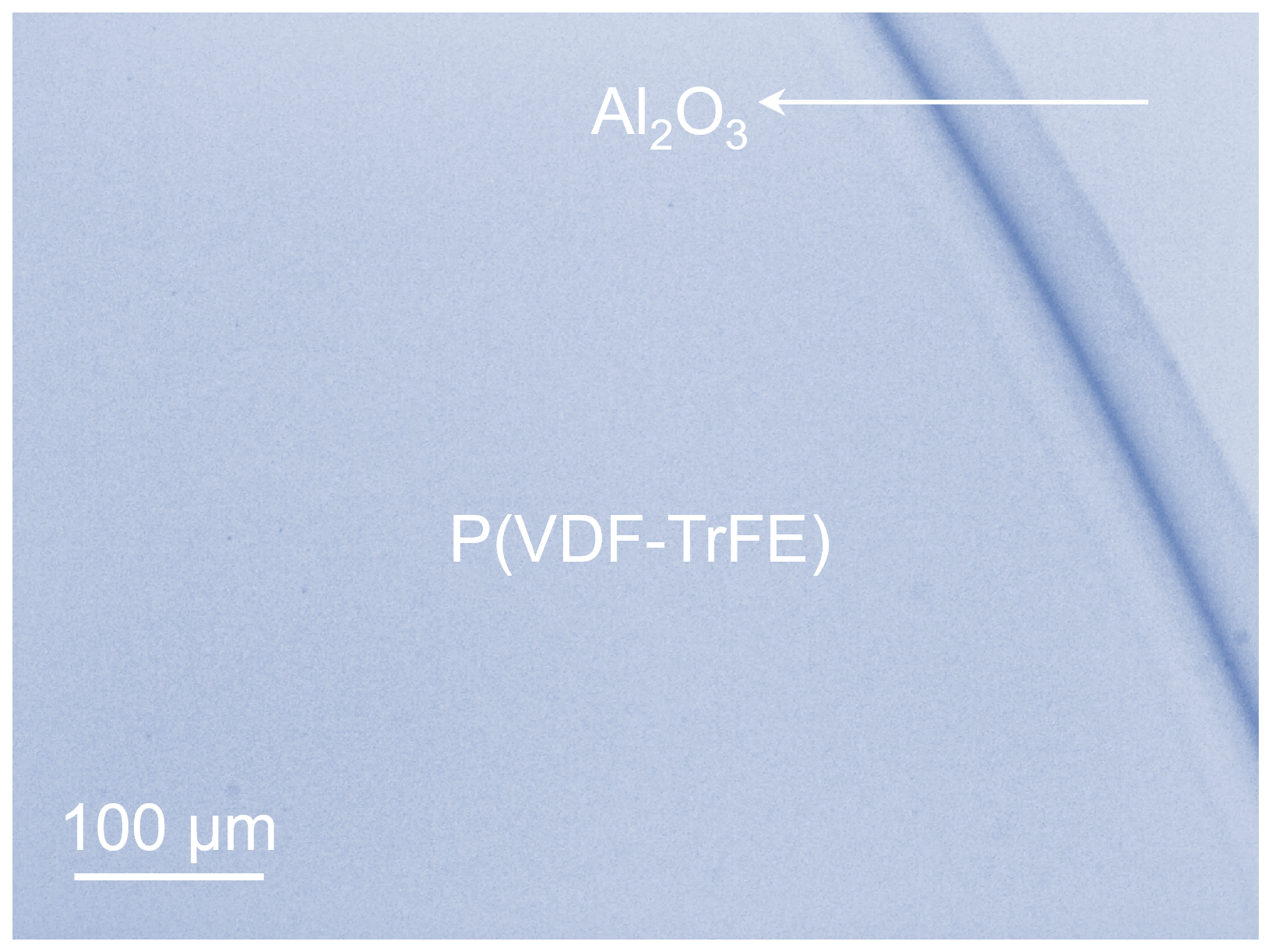


***Figure S2. Optical microscopy of the ultrathin P(VDF-TrFE).*** *The colour was modified to enhance the visibility.*


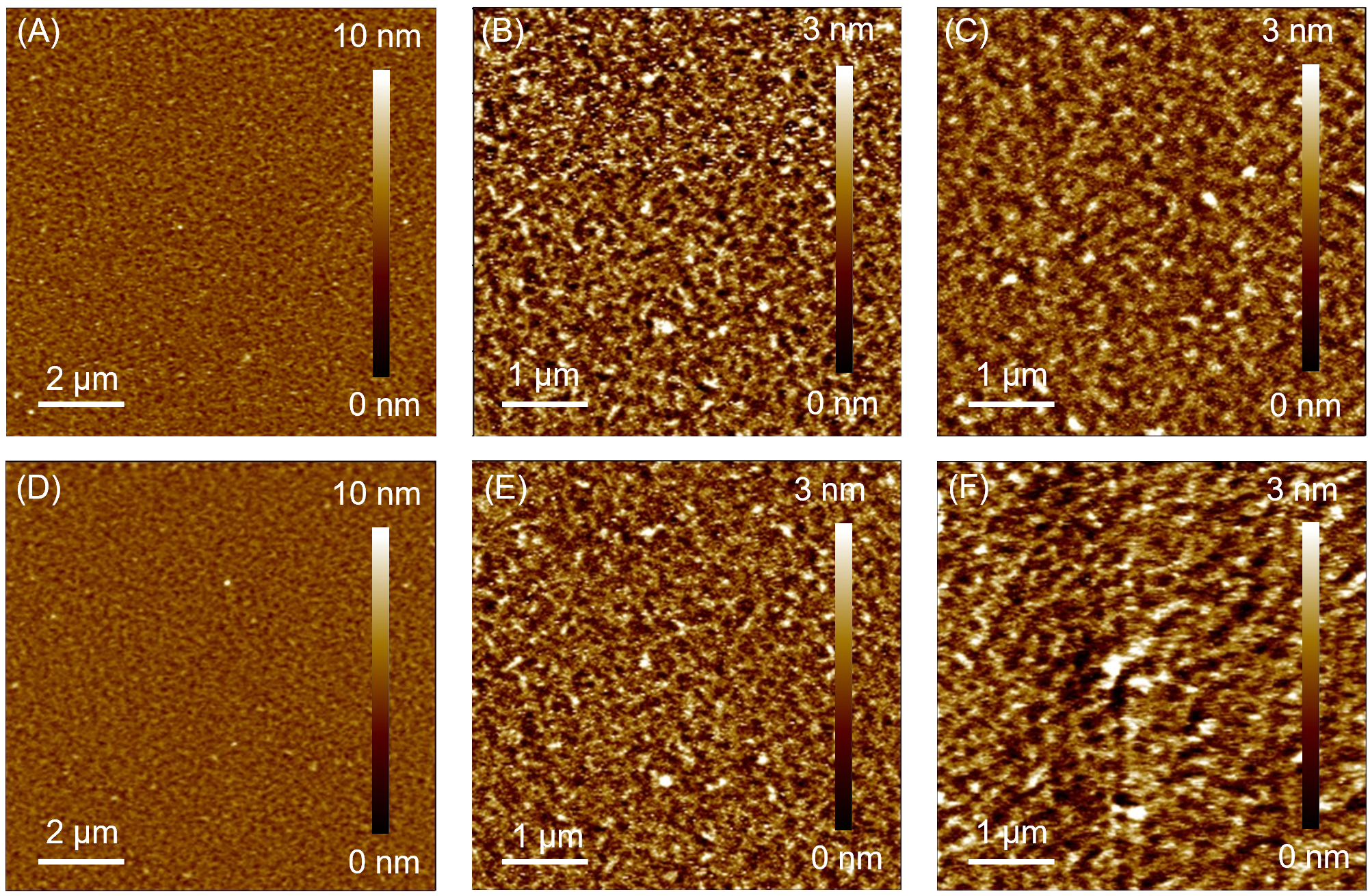


***Figure S3. AFM images of the ultrathin crystalline P(VDF-TrFE) film on Al_2_O_3_.*** *The root-mean-squared (RMS) roughness are all less than 1 nm (A: 0.68 nm; B: 0.63 nm; C: 0.49 nm; D: 0.55 nm; E: 0.53 nm; F: 0.65 nm).*


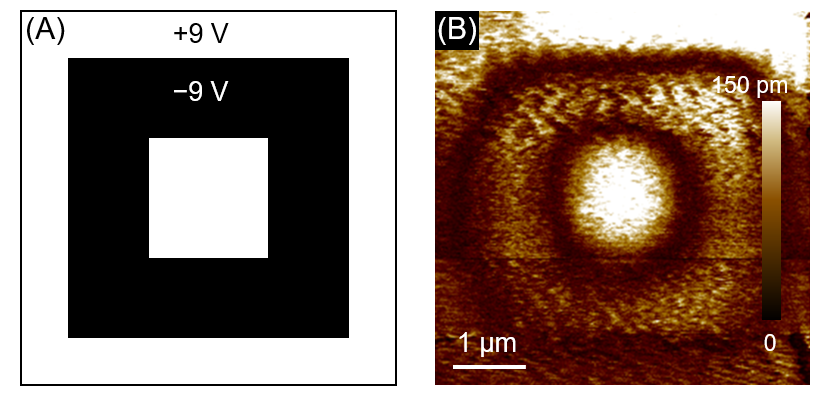


***Figure S4. Out-of-plane PFM measurements.*** *(A)* *The voltage definition of the scanning domain. (B) The amplitude image of the ultrathin P(VDF-TrFE) films.*


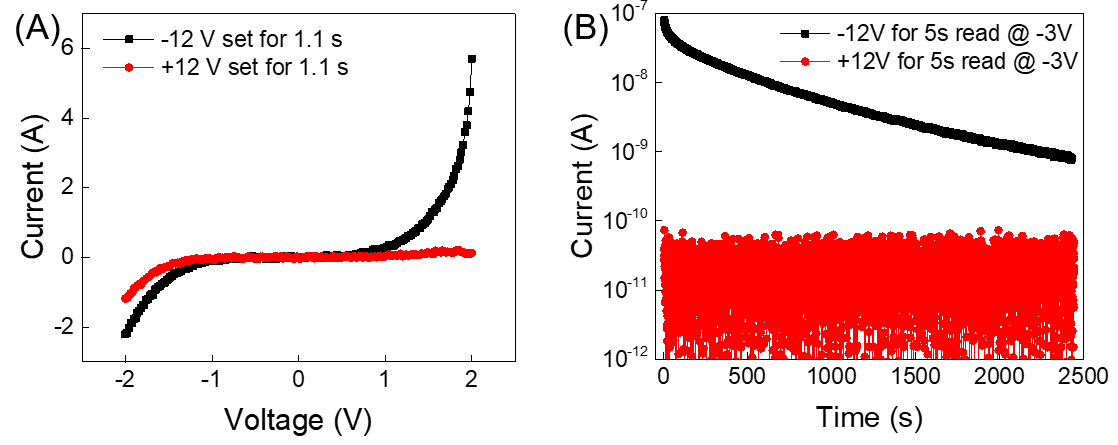


***Figure S5. The memory performance of the two-terminal planar organic ferroelectric devices.*** *(A) I–V curves in the low-voltage range after setting and resetting operations. (B) Retention measurements of the on/off currents.*


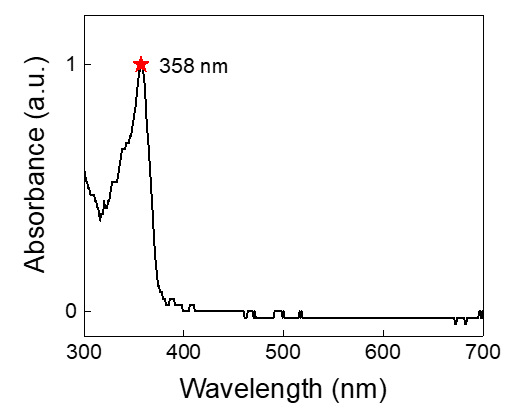


***Figure S6. Absorption spectra of C_8_-BTBT films on quartz.***


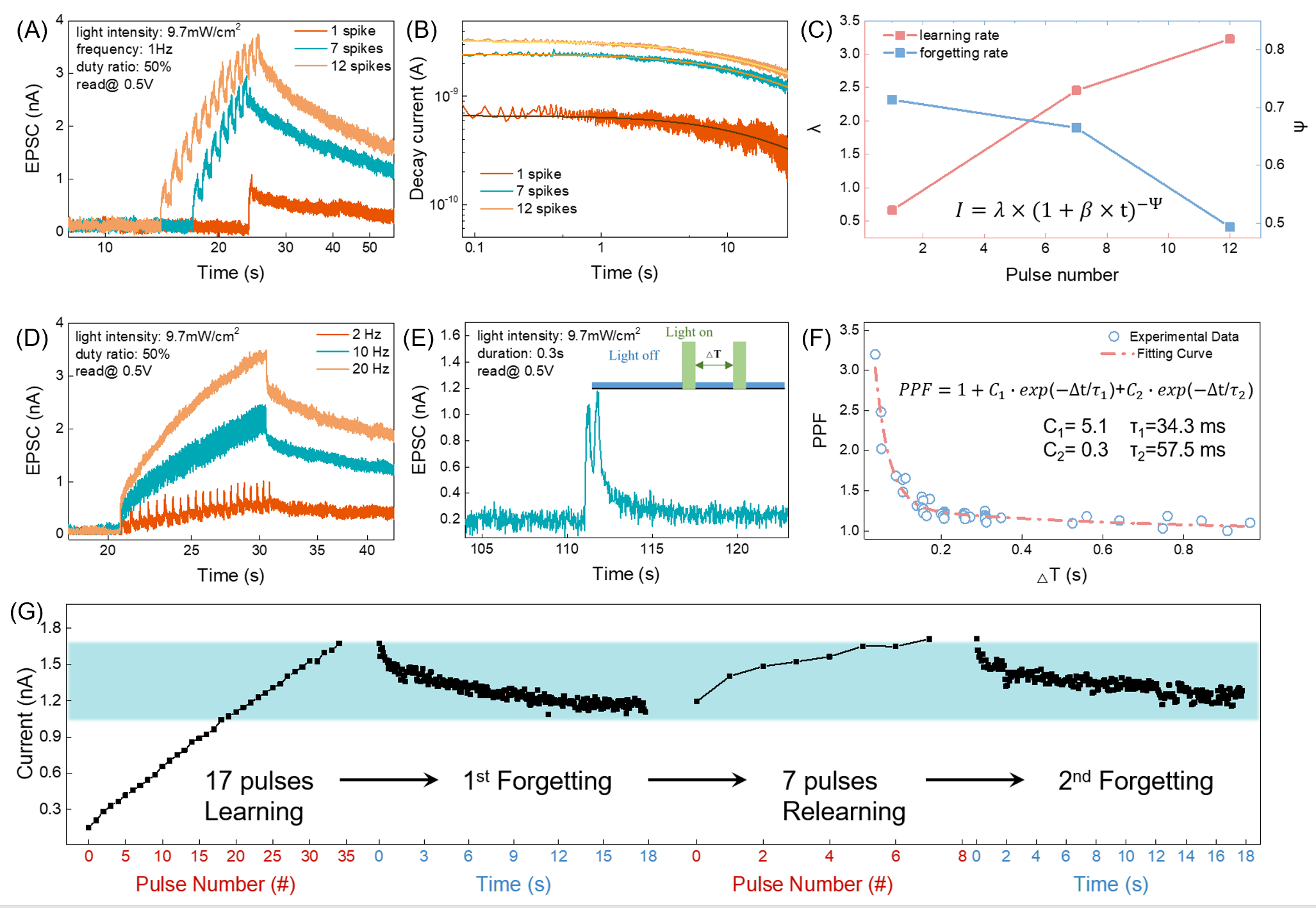


***Figure S7. An optically modulated organic artificial synapse under the downward polarization state of P(VDF-TrFE).*** *(A) The EPSC triggered by increasing the number of pulsed light stimuli. (B) Current decay is well fitted by the Wickel- gren’s power law. The data are taken from (A). (C) Changes in forgetting factor ψ and learning degree λ as the pulse number increases. (D) The EPSC triggered by increasing the frequency of pulsed light stimuli. (E) The EPSC triggered by a pair of pulses. (F) Paired-pulse facilitation (PPF) behaviours in the synaptic device. (G) “learning-experience” behaviour under pulsed light stimuli.*


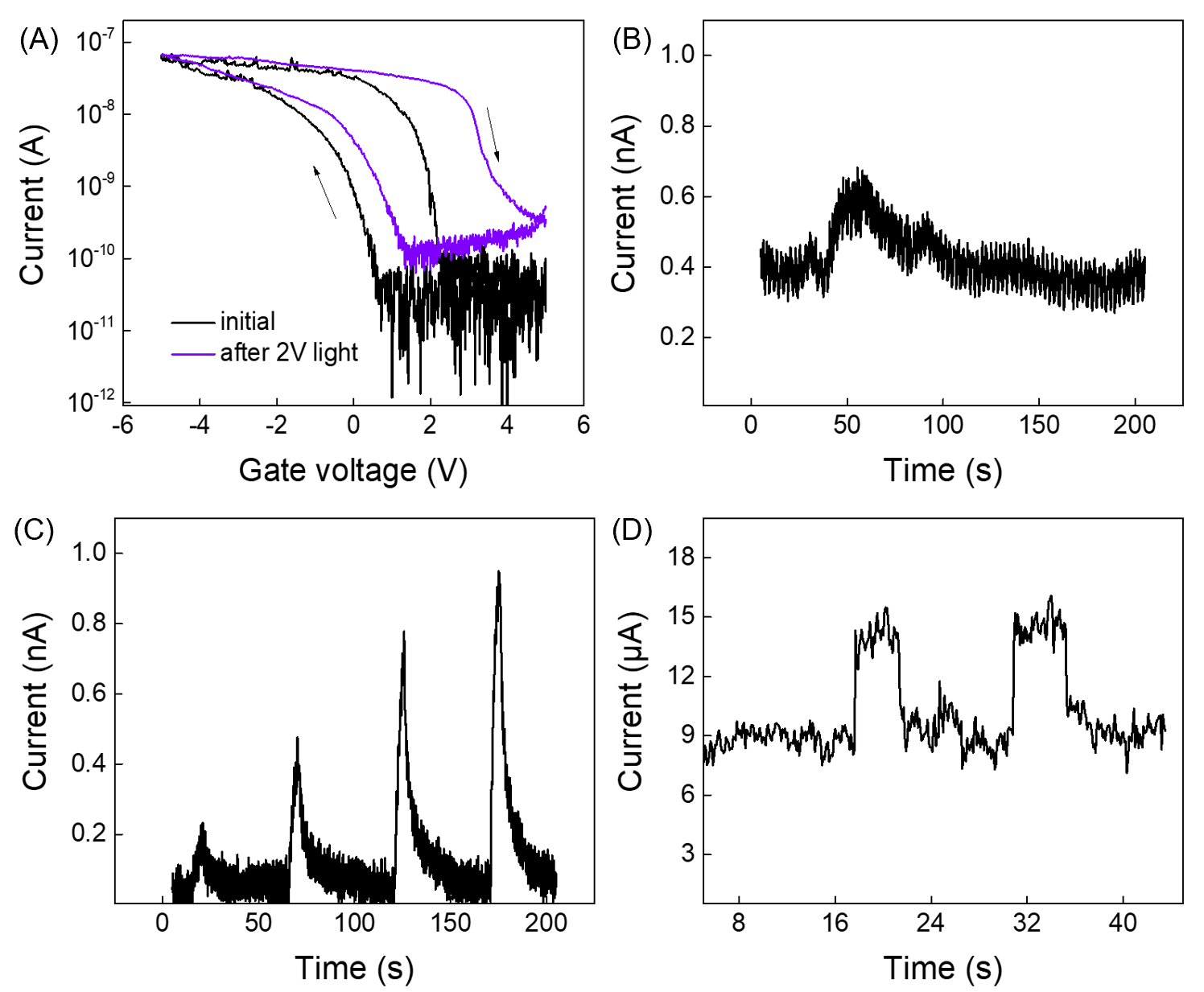


***Figure S8. The optoelectronic performance of the three-terminal organic ferroelectric devices.*** *(A) A typical I-V characteristic with a sweep range of ±5 V under dark (black) and after illumination (violet); (B) The source-drain current (I_ds_) triggered under illumination with the bottom gate floated; (C) The current triggered under illumination with the bottom gate of 5 V and (D) –5 V (V_ds_ = –0.1 V), exhibiting the characteristics of photoelectric synapses and photodetectors, respectively.*


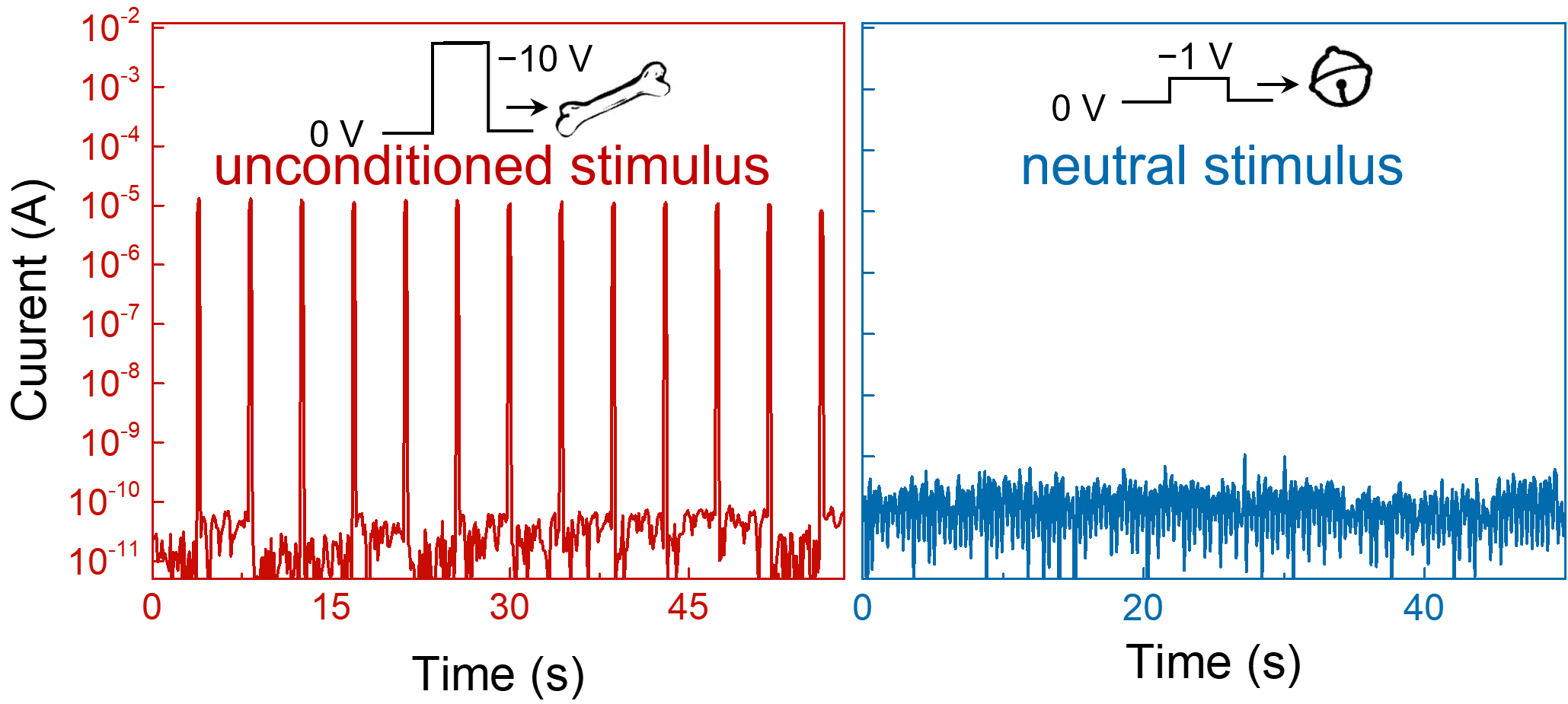


***Fig. S9. Unconditioned stimulus (US) and neutral stimulus (NS) and their current responses.*** *A US of a −10 V spike that produces an unconditioned response (~10 µA), while an NS of −1 V, causes a neutral response (~100 pA).*


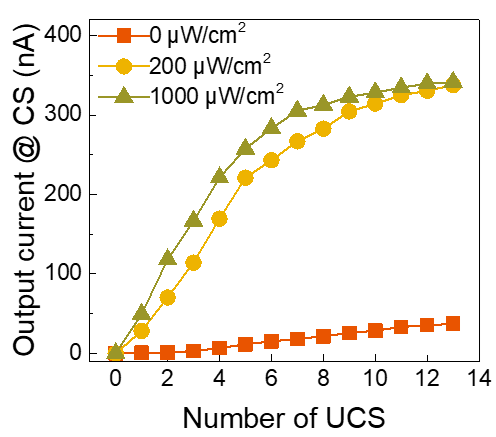


***Fig. S10. Quantitative analysis.*** *The output currents at CS (−1 V) depending on the number of US (−10 V) during the training processes.*

***Note1. Performance of a light-stimulated organic artificial synapse.***

Synaptic weight, or equivalently post-synaptic current, in the artificial synapse is increased when a pair of adjacent spikes is applied. In the oragnic ferroelectric neuromem, the excitons are continuously generated and separated under the light pulses. Electrons from the photo-generated excitons are trapped when the continued spikes are applied, inducing an increased excitatory post-synaptic current (EPSC). When the trapped charges are released, the EPSC will decrease distinctly. Biologically, synaptic capacity is modulated by changing the number of ions that escape to the synapses and accumulate there, which is confirmed by spike-number-dependent plasticity (SNDP). Here, different numbers of spikes were applied to change the synaptic weight in a device (fig. S7A). The power law of forgetting presented by Wickelgren in 1974 was used to describe the biological forgetting as

$I=\lambda\times{(1+\beta\times t)}^{-\Psi}$ (1)

where *I* is the memory strength, *t* is the decay time, *λ* is the state of long-term memory at *t* = 0 (i.e., degree of learning), *β* is a scale parameter, and ψ is the forgetting rate. All three of these forgetting processes are well quantified by the Wickelgren’s power-law model with different forgetting rates (fig. S7B). Using the factors of *λ* and *ψ*, the consolidation behavior can be described as follows: the deep learning (high *λ*) caused by repetitive learning can build up a more stable state which also decreases the forgetting (low *ψ*) (fig. S7C), in a way that is reminiscent of the consolidation function of neurons achieved by repeated learning. Meanwhile, the spike-rate-dependent plasticity (SRDP) was observed, in which the 0.9 nA peak current requires a 8 s duration for the 2 Hz stimulation but just 0.3 s for 20 Hz, indicating an enhanced weight under more frequent stimuli, producing a dynamic high-pass filter of the signal (fig. S7D). In addition, considering that the EPSC facilitation has a significant time interval (Δ*t*) dependence, the paired-pulse facilitation, PPF index (*A*_2_/*A*_1_∙100%) is applied, where *A*_2_ is the amplitude of the second current peak, and *A*_1_ is the amplitude of the first current peak. In fig. S7E, PPF is presented when a pair of adjacent spikes (Δ*t* = 0.5s; pulse width = 50 ms) is applied. As Δ*t* between two successive spikes increases, the facilitation effect weakened, which was well fitted by a biexponential Equation as

$PPF=1+C_{1}\cdot e^{-\frac{\Delta t}{\tau_{1}}}+C_{2}\cdot e^{-\frac{\Delta t}{\tau_{2}}}$ (2)

where *C*_1_ and *C*_2_ are the initial facilitation values of the two phases, and *τ*_1_ and *τ*_2_ are the characteristic relaxation times of the respective phases. The paramaters of the organic ferroelectric neuromem (*C*_1_ = 5.1, *C*_2_ = 0.3, *τ*_1_ = 34.3 ms, and *τ*_2_ = 57.5 ms), are similar to those in biological synapses (fig. S7F). Meanwhile, the interesting “learning-experience” behavior of human brains has also been demonstrated in fig. S7G. A human brain relearns a forgotten memory faster than a new memory; this ability has “time-saving” effects. In these studies, the amount of stimulation to attain the same synaptic weight was lower during relearning than during the first learning process. This result implies that memorization affects future learning processes and saves time. During the first learning process, 17 consecutive pulses were applied to get a certain level of synaptic weight, which was then allowed to decay spontaneously (i.e., to forget) over time of 18s. During the relearning process, only 7 consecutive pulses were required to obtain the specific synaptic weight that had been attained during the first learning process. This phenomenon showed that forgotten information can be relearned more easily than new information in artificial synapses, and this ability is similar to that of a biological brain.

*Table [S1]. Comparison with* *the electronic Pavlovian dogs in literature. RC and RN represent the conditional and neutral responses.*

| Materials system | Structure | R_C_ | R_N_ | Environmental tunability | Power consumption (J) | Ref. |
| --- | --- | --- | --- | --- | --- | --- |
| simulation | circuit | 8 V | 7 V | no | - | [1] |
| Pt/Ge0.3Se0.7/SiO_2_/Cu | memristor based circuit | −6.5 V | 6.5 V | no | 6.3 × 10^−3^ | [2] |
| pentacene:Au nanoparticle | transistor based circuit | 130 nA | 120 nA | no | 7.5 × 10^−5^ | [3] |
| NiOx | memristor based circuit | 0.83 V | 0.7 V | no | - | [4] |
| SmNiO_3_ | circuit | 1 kΩ | 2 kΩ | no | 0.4 | [5] |
| HfO_2_ | memristor based circuit | - | - | no | - | [6] |
| Ag/AgInSbTe/Ta | memristor based circuit | - | - | no | - | [7] |
| [PtMn(15)/CoFe(1.5)/Ru(0.85)/CoFeB(1.5)]: M2/MgO(0.6)/[CoFeB(1.5)]:M1 | magnetic tunnel junction based circuit | 1.4 kΩ | 2 kΩ | no | 1.8 × 10^−4^ | [8] |
| Pt/TiOx/TiO_2_/Pt | memristor based circuit | 4.5 V | 0.6 V | no | - | [9] |
| Ni/Nb-SrTiO_3_/Ti | memristor based circuit | 45 mV | 10 mV | no | - | [10] |
| Al_2_O_3_ nanoparticle:PI | memristor | 100 Ω | 100 kΩ | no | 1.3 | [11] |
| The Pt/Ag/SiO_x_:Ag/Ag/Pt diffusive memristor on a Pt/Ta_2_O_5_/TaO_x_/Pt capacitor | memristor and transistor based circuit | - | - | no | 1.4 × 10^−8^ | [12] |
| Chltosan | transistor | 1.2 μA | 530 nA | no | 4 × 10^−8^ | [13] |
| P_3_HT^*^/Ion-gel | transistors array | 50.8 μA | 5.8 μA | no | 2.5 × 10^−5^ | [14] |
| GaAs/AlGaAs | transistor based circuit | - | - | no | - | [15] |
| Ag/AgInSbTe/Ta | memristor based circuit | - | - | no | - | [16] |
| 0D-CdSe/ZnS QDs/2D-MoS_2_ | Phototransistor based circuit | 0.5 μA | 0.3 μA | no | 9.4 × 10^−8^ | [17] |
| PDVT-10^⁑^/PVP^⁂^:CsPbBr_3_ QDs. | transistor based circuit | 1.3 nA | 0.2 nA | no | 1.1 × 10^−6^ | [18] |
| Pd/carbon QDs/Ga_2_O_3_/Pt | memristor based circuit | - | - | no | - | [19] |
| W probe/IZO/nanogranular SiO_2_/ITO | junctionless electric-double-layer transistors | 100 μA | 20 μA | no | 7 × 10^−4^ | [20] |
| PEDOT:Tos/ PTHF^#^ composites | transistor based circuit | 17 μA | 8 μA | no | 1.2 × 10^−4^ | [21] |
| C_8_-BTBT/P(VDF-TrFE) | two-terminal planar nueromem | 100 nA | 0.1 nA | yes | 9.4 × 10^−6^ | this work |
| ^*^P_3_HT: poly(3-hexylthiophene); ^⁑^PDVT-10: poly[2,5-bis(2-decyltetradecyl)-pyrrolo[3,4- c]pyrrole-1,4(2H,5H)-dione-alt-5,5′-di(thiophen-2-yl)-2,2′-(E)-2- (2-(thiophen-2-yl)vinyl)thiophene]; ^⁂^pvp: poly(4-vinyl phenol); ^#^PEDOT:Tos/PTHF: poly(3,4-ethylenedioxythiophene): tosylate/Polytetrahydrofuran; | | | | | | |

References

[1] Y. V. Pershin and M. Di Ventra, “Experimental demonstration of associative memory with memristive neural networks,” Neural Networks, vol. 23, no. 7, pp. 881–886, 2010. (DOI: 10.1016/j.neunet.2010.05.001)

[2] M. Ziegler *et al.*, “An electronic version of Pavlov’s Dog,” Advanced Functional Materials, vol. 22, no. 13, pp. 2744–2749, 2012. (DOI: 10.1002/adfm.201200244)

[3] O. Bichler *et al.*, “Pavlov’s Dog Associative Learning Demonstrated on Synaptic-Like Organic Transistors,” Neural Computation, vol. 25, no. 2, pp. 549–566, 2013. (DOI: 10.1162/NECO_a_00377)

[4] S. G. Hu *et al.*, “Synaptic long-term potentiation realized in Pavlov’s dog model based on a NiO x -based memristor,” Journal of Applied Physics, vol. 116, no. 21, p. 214502, 2014. (DOI: 10.1063/1.4902515)

[5] S. D. Ha, J. Shi, Y. Meroz, L. Mahadevan, and S. Ramanathan, “Neuromimetic Circuits with Synaptic Devices Based on Strongly Correlated Electron Systems,” Physical Review Applied, vol. 2, no. 6, p. 064003, 2014. (DOI: 10.1103/PhysRevApplied.2.064003)

[6] S. G. Hu *et al.*, “Associative memory realized by a reconfigurable memristive Hopfield neural network,” Nature Communications, vol. 6, no. 1, p. 7522, 2015. (DOI: 10.1038/ncomms8522)

[7] Y. Li *et al.*, “Associative Learning with Temporal Contiguity in a Memristive Circuit for Large-Scale Neuromorphic Networks,” Advanced Electronic Materials, vol. 1, no. 8, p. 1500125, 2015. (DOI: 10.1002/aelm.201500125)

[8] D. I. Suh, J. P. Kil, Y. Choi, G. Y. Bae, and W. Park, “a Magnetic Tunnel Junction,” vol. 51, no. 11, pp. 52–55, 2015.

[9] L. Wang, H. Li, S. Duan, T. Huang, and H. Wang, “Pavlov associative memory in a memristive neural network and its circuit implementation,” Neurocomputing, vol. 171, pp. 23–29, 2016. (DOI: 10.1016/j.neucom.2015.05.078)

[10] Z. Tan *et al.*, “Pavlovian conditioning demonstrated with neuromorphic memristive devices,” Scientific Reports, vol. 7, no. 1, p. 713, 2017. (DOI: 10.1038/s41598-017-00849-7)

[11] C. Wu *et al.*, “Mimicking Classical Conditioning Based on a Single Flexible Memristor,” Advanced Materials, vol. 29, no. 10, p. 1602890, 2017. (DOI: 10.1002/adma.201602890)

[12] Z. Wang *et al.*, “Capacitive neural network with neuro-transistors,” Nature Communications, vol. 9, no. 1, p. 3208, 2018. (DOI: 10.1038/s41467-018-05677-5)

[13] F. Yu, L. Q. Zhu, H. Xiao, W. T. Gao, and Y. B. Guo, “Restickable Oxide Neuromorphic Transistors with Spike-Timing-Dependent Plasticity and Pavlovian Associative Learning Activities,” Advanced Functional Materials, vol. 28, no. 44, p. 1804025, 2018. (DOI: 10.1002/adfm.201804025)

[14] Y. Fu *et al.*, “Flexible Neuromorphic Architectures Based on Self-Supported Multiterminal Organic Transistors,” ACS Applied Materials & Interfaces, vol. 10, no. 31, pp. 26443–26450, 2018. (DOI: 10.1021/acsami.8b07443)

[15] P. Maier *et al.*, “Associative learning with Y-shaped floating gate transistors operated in memristive modes,” Applied Physics Letters, vol. 110, no. 5, p. 053503, 2017. (DOI: 10.1063/1.4975370)

[16] L. Wang and H. Zou, “A new emotion model of associative memory neural network based on memristor,” Neurocomputing, vol. 410, pp. 83–92, 2020. (DOI: 10.1016/j.neucom.2020.05.002)

[17] Y. Cheng *et al.*, “Vertical 0D‐Perovskite/2D‐MoS 2 van der Waals Heterojunction Phototransistor for Emulating Photoelectric‐Synergistically Classical Pavlovian Conditioning and Neural Coding Dynamics,” Small, vol. 2005217, p. 2005217, 2020. (DOI: 10.1002/smll.202005217)

[18] W. He *et al.*, “A multi-input light-stimulated synaptic transistor for complex neuromorphic computing,” Journal of Materials Chemistry C, vol. 7, no. 40, pp. 12523–12531, 2019. (DOI: 10.1039/c9tc03898a)

[19] Y. Pei, Z. Zhou, A. P. Chen, J. Chen, and X. Yan, “A carbon-based memristor design for associative learning activities and neuromorphic computing,” Nanoscale, vol. 12, no. 25, pp. 13531–13539, 2020. (DOI: 10.1039/D0NR02894K)

[20] C. Wan, J. Zhou, Y. Shi, and Q. Wan, “Classical conditioning mimicked in junctionless IZO electric-double-layer thin-film transistors,” IEEE Electron Device Letters, vol. 35, no. 3, pp. 414–416, 2014. (DOI: 10.1109/LED.2014.2299796)

[21] X. Ji *et al.*, “Mimicking associative learning using an ion-trapping non-volatile synaptic organic electrochemical transistor,” Nature Communications, vol. 12, no. 1, p. 2480, 2021. (DOI: 10.1038/s41467-021-22680-5)
